#  Efficacy of Generic Granisetron vs Kytril® for PONV in Major Gynecological Operations: A Randomized, Double-blind Clinical Trial 

**Published:** 2012

**Authors:** Ashraf Aleyasin, Somayeh Hanafi, Elham Saffarieh, Hassan Torkamandi, Sara Allahyari, Fariborz Sadeghi, Mohammadreza Javadi

**Affiliations:** a *Gynecology and Obstetrics Department, Dr. Shariati Hospital, Tehran University of Medical Sciences, Tehran, Iran. *; b *Pharmaceutical Care Department, Dr. Shariati Hospital, Tehran University of Medical Sciences, Tehran, Iran.*; c *Research Center for Rational Use of Drugs, Tehran University of Medical Sciences, Tehran, Iran. *

**Keywords:** Postoperative nausea and vomiting (PONV), Generic granisetron, Kytril®, Gynaecological surgery

## Abstract

Background Granisetron is a first-generation 5-HT3-receptor antagonist that has shown efficacy in preventing postoperative nausea and vomiting (PONV). In this randomized double-blind parallel-group clinical trial, we assessed the efficacy of generic granisetron versus Kytril®, in the prevention of PONV in patients undergoing general anesthesia for gynaecological surgeries. Method One hundred and twenty patients who were supposed to undergo major gynaecological surgeries (myomectomy and hysterectomy) in Dr. Shariati Teaching Hospital, Tehran, Iran were randomly assigned to either single dose generic granisetron (40 mcg/kg), or Kytril® (40 mcg/kg) at the end of the surgery. Two episodes of emetic symptoms (nausea and vomiting) were recorded by a gynaecologist who had no knowledge of which treatment each patient had received. This gynaecologist observed the patients at three different intervals: 6, 12 and 18 h post surgery. At the end of the observation period each patient evaluated the satisfaction with the study drug, and the gynaecologist evaluated sedation of the patients. Results In the generic granisetron group 47 and 13 patients, and in the Kytril® group 45 and 15 patients underwent hysterectomy and myomectomy respectively. No difference was observed between two treatment groups regarding postoperative nausea and vomiting control during 18 hours after the drugs administration. Also there were no differences in the satisfaction with the study drug between the generic granisetron and Kytril®. No difference in sedation scores was observed between two groups. Conclusion Generic granisetron exerts efficacy against PONV after gynaecological surgeries which is non-inferior to that of Kytril®.

## Introduction

Nausea and vomiting are among the most problematic symptoms experienced by patients after general anesthesia for major gynecological surgeries (1-3). Pharmacologic approaches for the prevention and treatment of postoperative nausea and vomiting (PONV), including butyrophenones (*e.g.*, droperidol), dopamine receptor antagonists (*e.g.*, metoclopramide) and anticholinergics (*e.g.*, scopolamine) have been reported (4-7). But undesirable adverse effects such as excessive sedation, hypertension, dry mouth, dysphoria and extra pyramidal symptoms have been noted (8-9).

With the introduction of newer and more expensive antiemetics (*e.g.*, 5-HT3-receptor antagonists), the controversy has arisen the most cost-effective antiemetic for routine prophylaxis (10-11). The 5-HT3-receptor antagonists are now the standard therapy for preventing PONV, since emesis is caused by the stimulation of 5-HT3-receptors located on vagal afferents by serotonin released from the enterochromaffin cells in the small intestine (12). Granisetron is a first-generation 5-HT3-receptor antagonist which shows considerable efficacy in reducing the incidence of PONV after chemotherapy(13) and various major surgeries (8, 14-17) specifically gynecological operations (4, 18).

Given the large cost of Kytril® compared with generic granisetron, healthcare costs might escalate with the use of Kytril®. However, if Kytril® was more effective than the generic granisetron, the increased cost might be justified. There are no studies comparing the efficacy of generic granisetron with Kytril®. Therefore, we conducted a randomized double-blind clinical trial to assess the efficacy of generic granisetron versus Kytril® for preventing PONV in female patients undergoing general anesthesia for major gynecological surgeries (myomectomy and hysterectomy).

## Experimental

From January 2011 till February 2010, 120 patients who were supposed to undergo the major gynecological surgeries (myomectomy and hysterectomy) in Dr. Shariati Teaching Hospital located in Tehran, Iran, were recruited for this randomized, double-blind clinical trial. This trial is registered in www.irct.ir, with IRCT201010134927N1 number. The approval of our institutional ethics committee and the informed consent was obtained from each patient. Patients who had gastrointestinal disease, the smokers, those who had a history of motion sickness, previous postoperative nausea and vomiting, or both, and those who had taken an antiemetic medication within 24 h before the operation, were excluded from the study.

No patients received preanesthetic medication. Anesthesia was induced with 0.1-0.3 μg/Kg intravenous sufentanil, 5 mg/Kg intravenous thiopentone, and 0.5 mg/Kg intravenous atracurium was used to facilitate tracheal intubation. When hemodynamic variables were stable, 1 mg/Kg lidocaine 2% was injected through the epidural catheter during the surgical procedure. Muscle relaxation was maintained with atracurium as required.

All study personnel were blinded to treatment assignment for the duration of the study, except for the drug assigner. Patients were randomly assigned to study groups according to a computer-generated table of random numbers (n = 60 each). Covariates known to affect emetic risk, such as sex, age, weight, surgery type and hospital stay were used as the stratification factors of minimization to ensure the balance between the treatment groups. At the end of the surgeries (myomectomy and hysterectomy), in a randomized double-blind manner, patients received a single dose of intravenously 40 μg/Kg generic granisetron (by Aburaihan Pharmaceutical Co.) or Kytril®(by Roche Pharmaceutical Co.). It has been demonstrated that 40 μg/Kg granisetron is the minimum effective dose for the prevention of PONV in patients undergoing the major gynecologic operations (19). It has also been shown that the efficacy of 40 μg/Kg granisetron is similar to that of 60 μg/Kg for the prevention of PONV, following the gynecological surgery. Higher doses may increase the undesirable adverse effects. Therefore, the dose of 40 μg/Kg chosen for this study, was within the clinically effective dose range (1). The patients were observed for the assessment of efficacy during 18 h after the drug administration. Two episodes of emetic symptoms (nausea and vomiting) were recorded by a gynecologist who had no knowledge of which treatment each patient had received. This gynecologist observed the patients at three different intervals: 6, 12 and 18 h after the surgery. *Nausea *was defined as an unpleasant feeling associated with the awareness of the urge to vomit, whereas *vomiting *was defined as the forceful expulsion of gastric contents from the mouth (20). At the end of the observation period, each patient evaluated the satisfaction with the study drug and the gynecologist evaluated the sedation of patients. The evaluations were performed on a linear numeric scale ranging from 0 (complete dissatisfaction) to 5 (complete satisfaction) and also from 0 (no sedation) to 5 (extreme sedation).

Statistical analyses of data between the treatment groups were performed by one-way analysis of variance (ANOVA), the chi-square test, the Fisher’s exact probability test (2-tailed), or the Mann-Whiney U-test as appropriate. A p-value < 0.05 was considered significant.

## Results

Patient demographic characteristics and types of operation were not different between the treatment groups (Table 1).

No difference was observed between the two treatment groups regarding the postoperative nausea and vomiting control during 18 h after the drugs administration (except for the nausea control at 6-12 h post myomectomy). In addition, there were no differences in the satisfaction of the study drug between the generic granisetron and Kytril®. No difference in sedation scores was observed between the two groups.

Table 2 compares generic granisetron and Kytril® regarding the types of gynecologic surgeries (myomectomy and hysterectomy).

**Table 1:** Patient demographic data

**p-value**	**Kytril**® **(n = 60)**	**Granisetron (n = 60)**	**Variable**	
0.064	43.4 9 26 21 4	46.7 5 22 24 9	*Age * Mean – yr Less than 35 yr 35-45 yr 45-55 yr More than 55 yr	
0.334	72.3	74.5	*Weight *(Mean) - kg	
0.800	2.5	2.3	*Hospital stay *(Mean) – day	
0.341	7/49	4/52	*Allergy existing *(Yes/No) - n	
0.693	45/15	47/13	*Surgery *(Hysterectomy/ Myomectomy) - n	
0.647	11 (18.6)	13 (22.0)	0-6 h – n (%)	*Vomiting*
0.407	6 (10.0)	9 (15.2)	6-12 h – n (%)	
0.380	5 (8.3)	8 (13.3)	12-18 h – n (%)	
0.256	19 (38.8)	21 (42.8)	0-6 h – n (%)	*Nausea*
0.275	11 (18.6)	16 (27.1)	6-12 h – n (%)	
0.852	11 (18.6)	12 (20.0)	12-18 h – n (%)	
0.202	56.3	63.8	*Sedation score *– Mean rank	
0.192	61.53	56.52	*Patient Satisfaction rating *– Mean rank	

There were no differences between the treatment groups

**Table 2:** Comparison of generic granisetron and Kytril® regarding the types of surgeries

**Myomectomy (n = 28)**			**Hysterectomy (n = 92)**			**Variable**	
**p-value**	**Granisetron (n = 15)**	**Kytril**® **(n = 13)**	**p-value**	**Granisetron (n = 45)**	**Kytril**® **(n = 47)**		
0.377	34.6	37.3	0.044	46.1	49.3	*Age *(Mean) - yr	
0.986	71.6	71.2	0.287	72.4	75.3	*Weight *(Mean) - kg	
0.321	1.8	4.3	0.186	2.7	1.7	*Hospital stay *(Mean) – day	
0.096	0/14	2/9	0.944	4/37	4/39	*Allergy existing *(Yes/No) - n	
0.250	4 (26.6)	2 (15.4)	0.748	8 (17.7)	9 (19.1)	0-6 h – n (%)	*Vomiting*
-	0 (0)	0 (0)	0.592	8(17.7)	6 (12.7)	6-12 h – n (%)	
-	0 (0)	0 (0)	0.231	8(17.7)	4 (8.5)	12-18 h – n (%)	
0.462	6 (40)	5 (38.4)	0.872	14 (31.1)	13 (27.6)	0-6 h – n (%)	*Nausea*
0.019	4 (26.6)	0(0)	0.952	11 (24.4)	11 (23.4)	6-12 h – n (%)	
0.345	0 (0)	1 (7.7)	0.848	11(24.4)	10 (21.2)	12-18 h – n (%)	
0.662	14.17	12.93	0.369	47.79	43.21	*Sedation score *– Mean rank	
0.083	11.50	14.38	0.533	43.93	46.09	*Patient Satisfaction rating *– Mean rank	

## Discussion

Post operative nausea and vomiting are still among the most common complications after the anesthesia and surgery, with a high incidence after the gynecologic operations (4). Many factors may influence the incidence of nausea and vomiting after the surgery performed under the general anesthesia. These factors include age, obesity, history of motion sickness, surgical procedure and anesthetic technique (1-2, 21). Granisetron is a 5-HT3-receptor antagonist which shows considerable efficacy in reducing the incidence of PONV after gynecologic operations.

The main finding of this clinical trial of patients undergoing two gynecologic surgeries is that the efficacy of generic granisetron in the prevention of nausea and vomiting is non-inferior to Kytril® during the 18 h period after the surgery. In the present study, two treatment groups were similar in terms of patient demographic characteristics and type of anesthesia and analgesic used post operatively. Therefore, the difference between the groups can be attributed to the difference in the generic granisetron and tested Kytril®. In this clinical trial, we demonstrated that the control of PONV during 18 h after the drug administration was identical between patients who had received generic granisetron or Kytril®. This suggests that antiemetic therapy with Kytril® is not superior to that with generic granisetron in the treatment of PONV in patients undergoing two gynecologic operations: myomectomy and hysterectomy. We could find no report comparing the efficacies of generic granisetron and Kytril® for the treatment of PONV after the operations.

The rationalization of health care expenditures has a high priority for the governments and the introduction onto the market of generic drugs produces notable savings (22-23). There seems to be a thought that although generic formulations are always less expensive than the corresponding brand-name drugs, they have not always the same effectiveness (24). The US Office of Generic Drugs (Center for Drug Evaluation and Research, FDA) addresses the problem of impurities that may occur in the manufacturing process of generic drugs (25). Some studies have reported variations in the efficacy of generic drugs compared with the corresponding brand-name drugs (26-28). These reports cause doubts about the interchangeability of generic and brand-name drugs. For instance, the results of a study by Crawford involving 2885 patients receiving antiepileptic treatment with phenytoin, carbamazepine, or valproic acid showed negative consequences associated with a shift to a generic formulation *e.g*., reappearance of convulsions and increases in social costs (29). Although the use of generic products may produce considerable savings, these savings should not be offset by increased hospitalization costs, nor should patients’ therapeutic stability be compromised. In other words, any loss of efficacy can have ethical and health repercussions, as well as important economic consequences (22).

On the other hand, Oles *et al. *and Silpakit *et al. *conducted randomized, double-blind studies in large patient populations and reported no difference in clinical efficacy of generic and brand carbamazepine (22). In another study by Dong *et al*, the four generic and brand-name levothyroxine preparations studied were bioequivalent by Food and Drug Administration criteria and were interchangeable in patients receiving thyroxine replacement therapy (30).

In Iran, Kytril® (US $ 14.5 for 3 mg (year of costing: 2011)) is much more expensive than the generic granisetron (US $ 2 for 3 mg (year of costing: 2011)). A cost-effectiveness ratio is a method of calculating the cost per unit of the benefit of a drug or other therapeutic interventions (31). In this study, a cost-effectiveness analysis was not performed. However, it is obvious that the use of the generic granisetron would dramatically decrease the cost of antiemetic therapy, assuming the similar hospital stay in both groups.

Implementing strategies such as those which require the patients to start with the most cost-effective drug therapy before moving to costlier medications, will reduce unnecessary use of expensive brand-name medications. Physicians’ fiduciary role of reducing the health care costs should not be ignored. Educating physicians about the use of generics will undoubtedly help to reduce these costs wherever possible. Promoting the use of generic medications should be a key part of patient management in a more cost-effective health care system (32).

We did not include a control group receiving placebo in our study. It has been suggested that if active drugs are available, placebo controlled trials may be unethical since PONV are very much distressing after major surgeries (8).
